# Evaluation of the Prognostic Value of Existing Scoring Systems for Nosocomial Infection in Patients with Decompensated Liver Cirrhosis

**DOI:** 10.5152/tjg.2022.21547

**Published:** 2023-01-01

**Authors:** Xu Zhao, Yu-ying Ou, Dan Guo, Xiao-qiong Che, Zi-qiong Li

**Affiliations:** Department of Infectious Disease, The First Affiliated Hospital of Chongqing Medical University, Chongqing, China

**Keywords:** Liver cirrhosis, nosocomial infection, predictive models

## Abstract

**Background:**

Many scoring systems have been developed to evaluate the severity and survival of end-stage liver disorder patients. However, the conduction of these different predicting models has not been thoroughly verified in cirrhotic patients with nosocomial infections. This study ended to compare the predictive accuracy of various scoring systems.

**Methods:**

During January 2015 and January 2020, liver cirrhosis patients with nosocomial infections were involved in this study. The clinical data, laboratory findings, and demographic characteristics of patients were collected during diagnosis. Patients were followed up for at least 6 months or till death.

**Results:**

One hundred thirty-one patients meeting the criteria were enrolled and followed up for at least 6 months. The mortality rate at 30 days, 3 months, and 6 months was 23%, 35.1%, and 39.6%, respectively. The univariate analysis showed that all scoring systems indicated statistical significance between the surviving group and the non-surviving group at 6 months. Model for end-stage liver disease-Na showed excellent predictive accuracy in predicting the survival at 30 days, 3 months, 6 months, with the area under the curve of 0.807, 0.850, and 0.844, respectively. Model for end-stage liver disease-Na demonstrated sensitivities of more than 85%. In contrast, the child-turcotte-pugh and albumin-bilirubin scores showed a poorer predictive capability.

**Conclusion:**

All 5 models for end-stage liver disease-related scores (model for end-stage liver disease, model for end-stage liver disease-to-serum sodium ratio, model for end-stage liver disease-Na, model for end-stage liver disease-Delta, snd integrated model for end-stage liver disease) exhibited a reliable prediction for mortality of long-term prognosis and short-term prognosis of cirrhotic patients with nosocomial infections. Among them, the model for end-stage liver disease-Na score might be the best choice.

**Main Points**

- All 5 models for end-stage liver disease (MELD)-related scores can reliably predict the short-term and long-term prognosis of nosocomial infection in patients with liver cirrhosis.
- Model for end-stage liver disease-Na had an excellent prediction accuracy and sensitivity of survival rates of more than 85% in predicting 30 days, 3 months, and 6 months, which may be the best choice.
- The proper use of model for end-stage liver disease-Na score will improve the quality of the clinical practice.

## Introduction

Cirrhosis is a leading reason for liver-associated death worldwide, with more than 1 million deaths annually.^1,2^ As a common complication of liver cirrhosis, infection remains a primary cause of incidence rate and mortality of patients with cirrhosis. More than a quarter of hospitalized patients with decompensated cirrhosis will find bacterial infection during admission or hospitalization,^3,4^ which will increase microbial resistance to antibiotics, bring additional economic burden, prolong hospital stay, and lead to long-term disability.^4-7^

Facing such a troublesome problem, what can we do? Risk assessment can minimize the negative impact of infection on patients with cirrhosis. At present, there are several scoring systems that can be used to predict the prognosis of patients with liver diseases, such as the child-turcotte-pugh (CTP) scoring system, albumin-bilirubin (ALBI) scoring system, model for end-stage liver disease (MELD) scoring system, platelet-albumin-bilirubin (PALBI) grading system, MELD-modified scoring systems (MELD-Na, integrated MELD [iMELD] score, and MELD to the serum sodium ratio [MESO]).^8-11^ Based on several specific clinical parameters, MELD scoring system and CTP scoring system are most widely used in the assessment of liver function. However, more and more studies indicated the limitations of the prediction accuracy of these evaluation systems.^12-14^ Model for end-stage liver disease,^15^ iMELD score,^16^ and MESO index^17^ were modified according to the original MELD scoring system for liver function and severity assessment. In addition, PALBI grade and ALBI grade were originally applied to evaluate the prognosis of patients with hepatocellular carcinoma. Recently, some studies have proven that ALBI score and PALBI score have certain predictive values for the prognosis of liver cirrhosis,^18-20^ hepatitis B virus (HBV)-related liver disordesr,^21^ acute-on-chronic liver failure (ACLF),^22^ cirrhosis-related upper gastrointestinal bleeding,^23,24^ and liver transplantation.^25^ AARC-ACLF score^26^ is considered to be a newly developed simple scoring system, which depends on Asian ACLF patients. A previous study^27^ showed that the AARC ACLF score was superior to other scoring systems in predicting the progression and prognosis of hospitalized patients with acute decompensated cirrhosis.

All scoring systems conduct differently in the clinical practice. However, the predictive value of these scoring systems for cirrhotic patients with nosocomial infections (NIs) is also unclear. Therefore, the purpose of this study was to verify the most accurate scoring system as the best prognostic scoring system for patients.

## Materials and Methods

This study has been approved by the Ethics Committee of The First Affiliated Hospital of Chongqing Medical University. Written informed consents were obtained from all patients involved in this study. 

### Patient Selection

According to the infection records and reports of patients in the nosocomial infection registration system, patients with liver cirrhosis admitted from January 2015 to January 2020 were included in this study. For patients with various NIs episodes during our study, this study only involved the first NIs.

Patients involved in this study were evaluated according to the following criteria: (1) the diagnosis of liver cirrhosis was based on laboratory examination, endoscopy, radiology, and clinical signs; (2) the patient was older than years old (≥18 years). The exclusion criteria were listed as the following: (1) hepatocellular carcinoma beyond the Milan criteria; (2) the extrahepatic malignancy; (3) liver transplantation; (4) the previous organ transplantation; (5) severe extrahepatic complications, such as severe trauma and heart diseases, pulmonary disorders, infarction, and intracerebral hemorrhage; (6) human immunodeficiency virus infection; (7) pregnancy.

### Definitions

The diagnosis of liver cirrhosis was based on the following criteria: (1) previously available histological findings, clinical and biological data, endoscopic evidence, and ultrasound or imaging findings; (2) exclusion of other potential diseases. The clinical manifestations of non-infectious liver decompensation in patients with liver cirrhosis, such as ascites, variceal bleeding, hepatorenal syndrome, and hepatic encephalopathy, were verified according to the diagnostic criteria described by the International Ascites Club and the European Association for the Study of the Liver. The diagnosis of co-infection in patients with liver cirrhosis was carried out according to the clinical examinations (laboratory and imaging examinations) and pathogenic microorganism detection, culture, and identification. The specific infection was defined as follows: (1) the diagnosis of spontaneous-bacterial peritonitis (SBP) was that the number of neutrophils in ascites was greater than 250 cells/mm^3^ (≥250 cells/mm^3^), with or without positive ascites culture; (2) urinary tract infection meant that the urine leukocyte count exceeded 15 cells in each high-power field, and the urine culture was positive with urinary irritation symptoms; (3) pneumonia (fever above 38°C), respiratory symptoms or infection signs (the body temperature below 35°C, and/or white blood cell amounts more than 12 000/mm^3^ or less than 4000/mm^3^); (4) bloodstream infection was referred as the growth of non-common skin contaminant from ≥1 blood cultures (BCs) and the growth of common skin contaminant from ≥2 BCs drawn on the separate sites; (5) other bacterial infections such as skin or soft tissue infections were diagnosed based on positive pathogen detection and culture. Nosocomial infection was defined as an infection that didn’t exist at the time of admission but occurred 2 days post-admission to a hospital or a healthcare facility.^28^

### Data Collection

This study collected demographic characteristics and clinical information from medical records, including gender, age, hospital admission history within 12 months, laboratory indicators, etiologies and complications of liver cirrhosis, invasive treatment, antibiotic therapy, bacterial distribution, and laboratory parameters. Demographic data were collected at the time of admission. For the laboratory parameters, including total bilirubin, international normalized ratio (INR), albumin (Alb), creatinine (Cr), C-reactive protein, leukocytes, platelets, arterial blood lactic acid, and procalcitonin, the worst parameter values during hospitalization were selected in this study. The prognostic models for predicting 30-day, 3-month, and 6-month mortality mainly included CTP, MELD, MELD-Na, iMELD, MESO, ALBI, PALBI, and AARC. All these models were calculated according to the published formulas. The models were evaluated weekly according to the latest clinical parameters and the worst value was selected for analysis. All patients were followed up for at least 6 months post-discharge from the hospital. Patients with competitive risk events unrelated to liver disease were further excluded from this study.

### Calculation of Scoring Systems

MELD score=11.2×log_e_ [INR]+9.57×log_e_[Cr]+3.78×log_e_ (bilirubin) + 6.4 × (etiology). In the above formula, for etiology, the value was 0 when cholestasis or alcoholism, otherwise it was 1. The lower bound of other 3 variables (INR, bilirubin, and Cr) was 1, while the upper bound of serum Cr was 4.

MELD-Na score=MELD-Na-[0.025 × MELD × (140-Na)] + 140. Here, the maximum of Na was 135 mEg/L and the minimum of Na was 120 mEq/L.

iMELD score = MELD + (age×0.3)-(0.7×Na+100).

MESO index = [MELD/Na (mEq/L)] × 100.

PALBI = (2.02×log_10_ bilirubin) + (-0.37× (log_10_ bilirubin)^2^) + (-0.04×albumin) + (-3.48×log_10_ platelets) + (1.01× (log_10_ platelets)^2^).

ALBI score = (log_10_ bilirubin (μmol/L) × 0.66) + (albumin(g/L) × 0.085).

The calculation of other scoring systems referred to the published formula.^8,26^ The 2 modified scores, including MELD-Delta and CTP-Delta, were the difference between the discharge score and the admission score.

According to the suggested cut-off points of PALBI, CTP, AARC, ALBI, and scores, patients were divided into 3 groups. The Child-Pugh-A score was defined as 5-6 points, Child-Pugh-B score was defined as 7-9 points, and Child-Pugh-C score was defined as 10-15 points. For ALBI score, grade 1 was defined as score less than −2.60 (<−2.60), grade 2 was defined as a score between −2.60 and −1.39, and grade 3 was defined as a score more than −1.39 (>−1.39). For PALBI score, grade 1 was defined as a score less than −2.53 (≤−2.53), grade 2 was defined as score between −2.52 and −2.09, and grade 3 was defined as score more than −2.09 (>−2.09). AARC ACLF score in this study was divided into three classes, including low risk (AARC ACLF <9), intermediate risk (9≤ AARC ACLF <12), and high risk (AARC ACLF ≥12).

### Statistical Analysis

The statistical analyses were carried out with Statistical Package for the Social Sciences 19.0 software (IBM Corp., Armonk, NY, USA). Continuous variables were presented as mean ± standard deviation (SD) or median (range) and compared using the Mann–Whitney *U* test. Categorical variables were presented as frequency (%) and compared using the chi-square test or Fisher’s exact test. In addition, we performed a receiver-operating characteristic curve (ROC) analysis. At the same, the area under the receiving-operator characteristics curves (AUROCs) was calculated with 95% CIs to evaluate the discrimination accuracy of the mortality scoring systems for cirrhotic patients with NIs. Delong test was used to compare differences among the prediction ability of scoring systems. Furthermore, we used the maximum specificity and sensitivity to ascertain the best cut-off point for scores. All statistical tests were 2-sided and *P* < .05 was considered to be statistically significant in this study.

## Results

### Patients and Diagnosed Diseases

**Figure 1.**
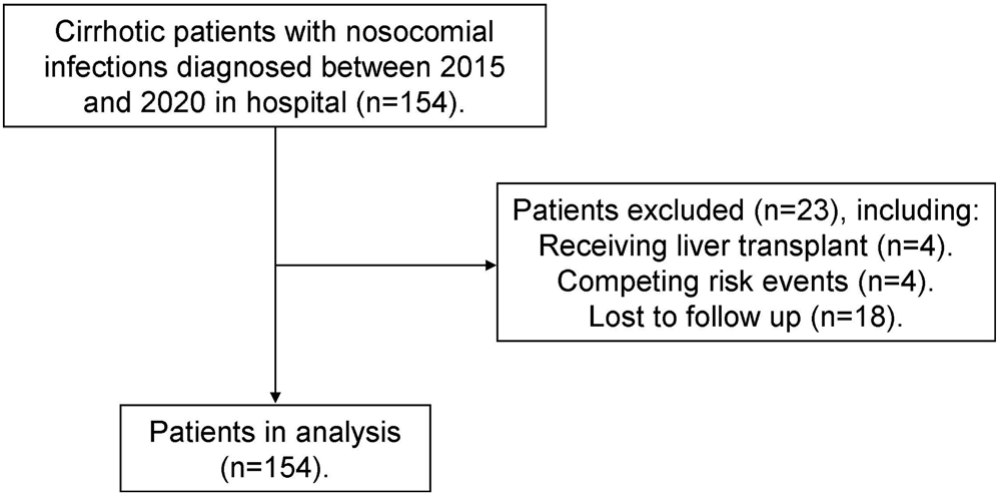
Flowchart for analysis of patients selected in this study.

A total of 131 consecutive liver cirrhosis patients with NIs were analyzed retrospectively (Figure 1). As shown in Table 1, the mean age of the patients was 54.9 ± 11.02 years, and most of them were men (n = 97, 73.4%). More than three-quarters of the patients (103, 78.63%) had HBV infection and 9.92% had alcoholic cirrhosis. About three-quarters (90.08%) of patients had ascites, followed by hepatic encephalopathy (29.77%) and variceal bleeding (25.95%). For comorbidities, diabetes mellitus accounted for 23.6%, followed by hypertension (19.85%) and chronic renal disease (9.16%).

### Grades of Consecutive Cirrhotic Patients

Most participants were ALBI grade 3 (75.7%), PALBI grade 1 (40.1), AARC grade 1 (54.5%), and CTP Class C (65.1%). The mean MELD, MELD-Delta, MELD-Na, iMELD, and MESO were 24.97 ± 10.35, 0.53 ± 8.43, 27.73 ± 12.85, 47.17 ± 11.21, and 1.86 ± 0.78, respectively.

### Grades of Consecutive Cirrhotic Patients

The overall mortality rate of participants at 30 days, 3 months, and 6 months was 22.7%, 34.8%, and 39.3%, respectively. According to their surviving status, we further divided them into the surviving group and the non-surviving group. Univariate analysis results showed (Table 2) that there were significant differences in the number of complications, leukocyte counts, total bilirubin, serum Cr, and INR between the 2 groups. In addition, there were significant differences in all scoring systems between 2 groups, except for the 30-day ALBI score.

### Accuracy for the Different Scoring Systems for Predicting the Survival

As shown in Table 3, all scoring systems had significant performance in predicting mortality (*P* < .001), except for ALBI (*P* = .261, .029, and .024, at 30 days, 3 months, and 6 months, respectively). The MELD-Na (AUC: 0.807) and CTP-Delta (AUC: 0.801) appeared to demonstrate significant predictive accuracy with AUC more 0.80 for patients with 30-day survival, and about 6 scoring systems’ AUC value ranged from 0.7 to 0.8, showing moderate predictive capability (iMELD: 0.794, MELD-Delta: 0.789, MESO: 0.784, MELD: 0.780, PALBI: 0.710, AARC: 0.726). For the survival status of 3-month and 6-month mortality, there were 5 and 4 scoring systems respectively had excellent accuracy in predicting cirrhosis complicating NIs, especially for the MELD-based scoring system. At 3-month mortality, the AUROCs for MELD-Na score, MELD-Delta score, iMELD score, MESO score, and MELD score were 0.850, 0.840, 0.836, 0.834, and 0.832, respectively. When predicting mortality at 6-month, MELD-Na, iMELD, MESO, and MELD indicated significantly predictive performance, and AUROCs were 0.844, 0.835, 0.834, and 0.832, respectively. However, AUROCs value of ALBI score at 30-days, 3-month, and 6-month was 0.568, 0.616, and 0.617, respectively. AUROCs value of CTP score at 30-days, 3-month, and 6-month was 0.640, 0.653, and 0.648, respectively. The AUROCs value of CTP-Delta was 0.691 at 6-month. In addition, the sensitivity of ALBI score at 30-days, 3-month, and 6-month was 86.67%, 91.3%, and 90.38%, whereas the specificity was 26.73%, 31.76%, and 32.91%, respectively.

**Figure 2.**
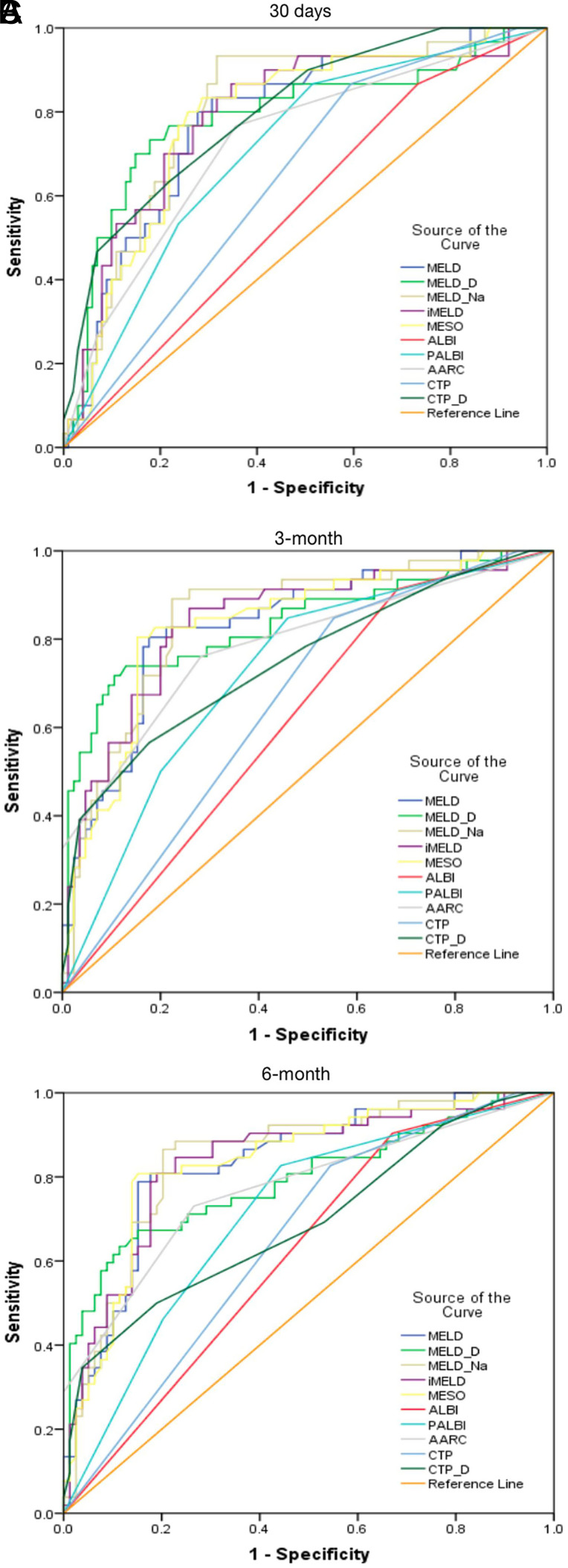
AUROCs value of ALBI score and CTP score at 30-day (A), 3-month (B), and 6-month (C). ALBI, albumin-bilirubin; CTP, child-turcotte-pugh score; AUROCs, areas under receiving-operator characteristics curves.

The MELD-Na showed its outstanding performance in predicting 30-days, 3-month, and 6-month survival (with AUC of 0.807, 0.850, and 0.844, respectively). Using the best MELD-Na cut-off value (>27.68), the prediction sensitivity and specificity of 30-days and 3-month mortality were as high as 93.33%, 68.32% and 89.13%, 77.65%, respectively. Additionally, both iMELD score and MESO score had high accuracy in predicting 3-month mortality and 6-month mortality (AUC>0.830). All 5 MELD-dependent models were significantly better than ALBI score or CTP score in predicting mortality. As shown in Table 3, the AUROCs values of ALBI score and CTP score at 30-days (Figure 2A), 3-month (Figure 2B), 6-month (Figure 2C), and CTP-Delta score at 6-month ranged from 0.5 to 0.7, which indicated poor prediction ability.

## Discussion

Hyponatremia with sodium (+) (Na+) levels less than 135 mmol/L, is considered to be the most common electrolyte disorder in the clinic. Hyponatremia is usually caused by visceral and systemic vasodilation, which is the reduction of effective arterial blood volume, resulting in excessive impermeable secretion of antidiuretic hormone.^29^ A previous study^30^ published in 2005 showed that hyponatremia may increase the mortality of patients with liver cirrhosis by 7 folds within 3 months (3-month), which has aroused great concern. In recent years, more and more studies^31-34^ have confirmed that serum sodium is closely related to the poor prognosis of patients with a bacterial infection.

Some prognostic scores combined with serum sodium concentration, including MELD-Na score, iMELD, and MESO index, showed good prognostic ability in patients with liver cirrhosis.^35,36^ Similarly, ROC analysis in our study also showed that iMELD, MELD-Na, MESO indexes were better than other prognostic scoring systems, especially in the prediction of 3-month and 6-month prognosis.

A recent study by Fricker et al^37^ showed that MELD-Na was closely associated with short-term mortality for cirrhotic patients with NIs (OR: 5.7, 95% CI: 5.47-5.91, *P* < .001). Moreover, in another study,^38^ multivariate analysis showed that MELD-Na score was an independent indicator of prognosis in patients with liver cirrhosis with skin tissue and soft tissue infection (with OR of 1.15 and 95% CI of 1.06-1.25). This study is consistent with the results of Fricker et al^37^ showing that MELD-Na indicated a prominent predictive value in cirrhotic patients with NIs in terms of short-term or long-term prognosis. In accordance with other studies (AUC: 0.7-0.92),^36,39,40^ the AUC of MELD-Na in this study was greater than 0.80. The multivariate analysis in a previous study^41^ also showed that MELD-Na score in patients with liver cirrhosis complicated with SBP was an independent prognostic predictor, and its haphazard ratio (mortality) was 1.09 (*P* < .001).

Unlike other previous studies, ROC analysis in this study showed CTP score and ALBI score were not enough to predict the prognosis of cirrhotic patients with NIs (AUC: 0.5-0.7), especially in short-term prognostic prediction. A systematic review^42^ showed that ALBI was more effective in predicting prognosis and overall survival of patients than the CTP score. In contrast, in this study, the predictive ability of the CTP score was better than that of the ALBI score.

This study also demonstrated some limitations. First, this study was a single-center study and the number of patients included was relatively small. Second, the etiological distribution of included patients with liver cirrhosis was an imbalance because more than 70% were HBV-related liver cirrhosis. Therefore, a large cohort study of stratified cirrhosis cases for different causes will be discussed to further confirm the findings of this study. Third, the occurrence time of NIs was often unable to be accurately determined, with might lead to potential bias in the study. Additionally, different treatment strategies for patients with NIs might also affect the prognosis of patients.

In conclusion, MELD-Na showed excellent performance in evaluating the prognosis of patients with liver cirrhosis complicated with NIs. The proper use of MELD-Na score will improve the quality of clinical practice.

## Figures and Tables

**Table 1.** Baseline Characteristics of Patients

**Characteristics**	**Values**	**Characteristics**	**Values**
Age, mean ± SD (years)	**54.9 ± 11.02**	MELD (mean ± SD)	**24.97 ± 10.35**
Gender, n (%):		MELD-Delta (mean ± SD)	**0.53 ± 8.43**
Male	**97 (73.4)**	MELD-Na (mean ± SD)	**27.73 ± 12.85**
Female	**34 (25.95)**	iMELD (mean ± SD)	**47.17 ± 11.21**
Etiology, n (%):		MESO (mean ± SD)	**1.86 ± 0.78**
Hepatitis B	**103 (78.63)**	ALBI Score, n (%)	
Hepatitis C	**6 (4.58)**	Grade 1 (≤−2.60)	**1 (0.7)**
Alcohol	**13 (9.92)**	Grade 2 (>−2.60 to −1.39)	**30 (22.7)**
Autoimmune	**4 (3.05)**	Grade 3 (>−1.39)	**100 (75.7)**
Cryptogenic	**1 (0.76)**	PALBI score, n (%)	
PBC	**2 (1.53)**	Grade 1 (≤−2.53)	**53 (40.1)**
Other	**2 (1.53)**	Grade 2 (−2.53 to −2.09)	**38 (28.7)**
The number of complications, n (%):		Grade 3 (>−2.09)	**40 (30.3)**
≤3	**104 (78.7)**	AARC, n (%)	
>3	**27 (20.4)**	Grade 1 (5-7)	**72 (54.5)**
Complications, n (%):		Grade 2 (8-10)	**44 (33.3)**
Hepatic encephalopathy	**39 (29.77)**	Grade 3 (11-15)	**15 (11.3)**
Ascites	**118 (90.08)**	CTP, n (%)	
Variceal bleeding	**34 (25.95)**	Class A	**6 (4.5)**
Hepatorenal syndrome	**19 (14.50)**	Class B	**39 (29.5)**
Diabetes mellitus, n (%)	**31 (23.66)**	Class C	**86 (65.1)**
Hypertension, n (%)	**26 (19.85)**	CTP-Delta ≥0 <0	**(78 [59.5])** **27 (53[40.5])**
Chronic renal disease, n (%)	**12 (9.16)**	Survival at 30 days, n (%)	
Laboratory results, median(IQR)/ mean±SD		Alive	**101 (77)**
Leukocyte, [10^9^/L]	**7.85 (5.48-11.9)**	Death	**30 (23)**
Hemoglobin, [g/L]	**2 (1-2)**	Survival at 3-month, n (%)	
Platelets, [10^9^/L]	**54 (36-91)**	Alive	**85 (64.9)**
Total bilirubin, [µmol/L]	**254.61 ± 207.24**	Death	**46 (35.1)**
Albumin, [g/dL]	**28.48 ± 4.62**	Survival at 6-month, n (%)	
Serum creatinine, [µmol/L]	**80 (65-122)**	Alive	**79 (60.4)**
INR, median (IQR)	**1.81 (1.49-2.45)**	Death	**52 (39.6)**

PBC, primary biliary cholangitis; INR, international normalized ratio; MELD, model for end-stage liver disease; ALBI, albumin-bilirubin; CTP, child-turcotte-pugh score; PALBI, platelet-albumin-bilirubin grade; AARC, APASL ACLF Research Consortium score; MESO, MELD to serum sodium ratio; iMELD, integrated MELD score; MELD-Na, model for end-stage liver disease with serum sodium concentration.

**Table 2.** Comparison of Clinical Characteristics Between Survivors and Non-Survivors

**Variable**	**30-days**				**3-month**				**6-month**			
	**Survivor (n = 101)**	**Non-survivor (n = 30)**	***t*** **/Z/χ ^2^**	***P***	**Survivor (n = 85)**	**Non-survivor** **(n = 46)**	***t*** **/Z/χ ^2^**	***P***	**Survivor (n = 79)**	**Non-survivor (n = 52)**	***t*** **/Z/χ ^2^**	***P***
Gender, n (%)												
Male	74 (76.29)	23 (23.71)	0.139	.709	62 (63.92)	35 (36.08)	0.154	.695	57 (58.76)	40 (41.24)	0.371	.542
Female	27 (79.41)	7 (20.59)			23 (67.65)	11 (32.35)			22 (64.71)	12 (35.29)		
Age, n (%)												
20-40	10 (76.92)	3 (23.08)	0.405	.817	7 (53.85)	6 (46.15)	1.276	.528	7 (53.85)	6 (46.15)	0.312	.855
40-60	60 (78.95)	16 (21.05)			52 (68.42)	24 (31.58)			47 (61.84)	29 (38.16)		
>60	31 (73.81)	11 (26.19)			26 (61.90)	16 (38.10)			25 (59.52)	17 (40.48)		
Hospital stays, n (%), [days]												
≤30	56 (80.00)	14 (20.00)	0.716	.397	54 (77.14)	16 (22.86)	9.913	.002	50 (71.43)	20 (28.57)	7.77	.005
>30	45 (73.77)	16 (26.23)			31 (50.82)	30 (49.18)			29 (47.54)	32 (52.46)		
Organ support treatment, n (%)	41 (67.21)	20 (32.79)	6.319	.012	31 (50.82)	30 (49.18)	9.913	.002	26 (42.62)	35 (57.38)	14.911	<.001
Number of complications, n (%)												
≤3	86 (82.69)	18 (17.31)	8.94	.003	78 (75.00)	26 (25.00)	22.657	<.001	73 (70.19)	31 (29.81)	20.605	<.001
>3	15 (55.56)	12 (44.44)			7 (25.93)	20 (74.07)			6 (22.22)	21 (77.78)		
Complications, n (%):												
Hepatic encephalopathy	21 (53.85)	18 (46.15)	17.006	<.001	15 (38.46)	24 (61.54)	17.018	<.001	15 (38.46)	24 (61.54)	11.069	.001
Ascites	89 (75.42)	29 (24.58)	/	.296	73 (61.86)	45 (38.14)	/	.033	67 (56.78)	51 (43.22)	6.175	.013
Variceal bleeding	22 (64.71)	12 (35.29)	3.994	.046	18 (52.94)	16 (47.06)	2.875	.09	17 (50.00)	17 (50.00)	2.037	.154
Hepatorenal syndrome	9 (47.37)	10 (52.63)	/	.002	5 (26.32)	14 (73.68)	14.51	<.001	5 (26.32)	14 (73.68)	10.725	.001
Etiology, n (%):												
Hepatitis B	75 (72.82)	28 (27.18)	5.008	.025	60 (58.25)	43 (41.75)	9.306	.002	56 (54.37)	47 (45.63)	7.094	.008
Hepatitis C	6 (100.00)	0 (0.00)	/	.336	5 (83.33)	1 (16.67)	/	.665	3 (50.00)	3 (50.00)	/	.681
Alcohol	12 (92.31)	1 (7.69)	/	.296	12 (92.31)	1 (7.69)	/	.033	12 (92.31)	1 (7.69)	6.175	.013
Autoimmune	4 (100.00)	0 (0.00)	/	.573	4 (100.00)	0 (0.00)	/	.297	4 (100.00)	0 (0.00)	/	.151
Cryptogenic	0 (0.00)	1 (100.00)	/	.229	0 (0.00)	1 (100.00)	/	.351	0 (0.00)	1 (100.00)	/	.397
PBC	2 (100.00)	0 (0.00)	/	>.999	2 (100.00)	0 (0.00)	/	.541	2 (100.00)	0 (0.00)	/	.518
Other	2 (100.00)	0 (0.00)	/	>.999	2 (100.00)	0 (0.00)	/	.541	2 (100.00)	0 (0.00)	/	.518
Leukocyte, median (IQR) [10^9^/L]	6.85 (5.06,10.24)	11.82 (8.98,19.43)	4.634	<.001	6.71 (5.06,9.52)	10.24 (7.9,15.24)	4.487	<.001	6.7 (5.06,9.46)	10.24 (7.88,14.59)	4.288	<.001
Hemoglobin, median (IQR) [10^9^/L]	2 (1,2)	2 (2,2)	1.527	.127	2 (1,2)	2 (2,2)	1.344	.179	2 (1,2)	2 (2,2)	1.487	.137
Platelets, median (IQR) [10^9^/L]	54 (36,90)	56 (34,100)	−0.079	.937	54 (36,91)	56.5 (35,90)	<0.001	>.999	54 (36,91)	56.5 (34.5,91.5)	0.024	.981
Total bilirubin, mean±SD [umol/L]	223.31 ± 204.2	359.96 ± 183.99	−3.289	.001	181.48 ± 158.18	389.73 ± 220.47	−5.666	<.001	171.91 ± 148.63	380.23 ± 221.39	−5.959	<.001
Albumin, mean±SD [g/dL]	28.47 ± 4.71	28.53 ± 4.38	−0.07	.944	28.48 ± 4.71	28.48 ± 4.52	0.005	.996	28.58 ± 4.79	28.33 ± 4.4	0.308	.758
Serum creatinine, median (IQR) [μmol/L]	74 (64,101)	118.5 (79,213)	2.786	.005	72 (63,86)	104 (79,193)	3.627	<.001	72 (63,86)	104 (72,183.5)	3.663	<.001
INR, median (IQR)	1.69 (1.48,2.13)	2.43 (1.78,3.19)	3.498	<.001	1.66 (1.42,2.03)	2.56 (1.71,3.19)	4.911	<.001	1.63 (1.38,1.99)	2.4 (1.71,3.15)	5.13	<.001
Serum sodium, median (IQR) [mmol/L]	141 (139,143)	140 (137,141)	0.958	.344	141 (139,143)	140 (137,142)	1.059	.293	141 (139,143)	140 (137.5,142.5)	1.186	.239
MELD	22.69 ± 9.64	32.64 ± 8.98	−5.037	<.001	20.77 ± 8.24	32.72 ± 9.39	−7.545	<.001	20.32 ± 8.23	32.02 ± 9.24	−7.573	<.001
MELD-Delta	−1.27 ± 7.69	6.59 ± 8.09	−4.853	<.001	−2.79 ± 6.55	6.66 ± 8.13	−7.231	<.001	−2.79 ± 6.7	5.58 ± 8.33	−6.341	<.001
MELD-Na	24.74 ± 11.72	37.8 ± 11.45	−5.385	<.001	22.41 ± 10.07	37.57 ± 11.65	−7.779	<.001	21.99 ± 10.2	36.46 ± 11.55	−7.533	<.001
iMELD	44.6 ± 10.33	55.82 ± 9.76	−5.286	<.001	42.52 ± 9.13	55.76 ± 9.55	−7.791	<.001	42.06 ± 9.14	54.94 ± 9.52	−7.766	<.001
MESO	1.69 ± 0.73	2.43 ± 0.68	−5.001	<.001	1.54 ± 0.62	2.44 ± 0.71	−7.529	<.001	1.51 ± 0.62	2.39 ± 0.69	−7.556	<.001
ALBI Score	3 (2,3)	3 (3,3)	1.52	.129	3 (2,3)	3 (3,3)	2.96	.003	3 (2,3)	3 (3,3)	3.067	.002
PALBI	2 (1,2)	3 (2,3)	3.715	<.001	1 (1,2)	2.5 (2,3)	4.505	<.001	1 (1,2)	2 (2,3)	4.33	<.001
AARC	1 (1,2)	2 (2,3)	4.206	<.001	1 (1,2)	2 (2,3)	6.031	<.001	1 (1,2)	2 (1,3)	5.868	<.001
CTP	3 (2,3)	3 (3,3)	2.798	.005	3 (2,3)	3 (3,3)	3.464	.001	3 (2,3)	3 (3,3)	3.436	.001
CTP-Delta	0 (−1,0)	1 (0,2)	5.092	<.001	−1 (−1,0)	1 (0,2)	4.803	<.001	0 (−1,0)	0.5 (−1,2)	3.775	<.001

*P* value <.05 is considered significant.

PBC, primary biliary cholangitis; INR, international normalized ratio; MELD, model for end-stage liver disease; ALBI, albumin-bilirubin; CTP, child-turcotte-pugh score; PALBI, platelet-albumin-bilirubin grade; AARC, APASL ACLF Research Consortium score; MESO, MELD to serum sodium ratio; iMELD, integrated MELD score; MELD-Na, model for end-stage liver disease with serum sodium concentration.

**Table 3.** Accuracy of Different Scoring Systems in Predicting the Survival of Patients

**Prognostic score**	**AUROC**	***P***	**Cut-off point**	**Sensitivity**	**Specificity**	**PLR**	**NLR**
***30-day***							
*MELD*	0.780	.000	25.91	83.33%	69.31%	2.72	0.24
*MELD-Delta*	0.789	.000	2.99	73.33%	82.18%	4.11	0.32
*MELD-Na*	0.807	.000	27.68	93.33%	68.32%	2.95	0.10
*iMELD*	0.794	.000	46.50	86.67%	65.35%	2.50	0.20
*MESO*	0.784	.000	1.97	83.33%	71.29%	2.90	0.23
*ALBI*	0.568	.261	2.00	86.67%	26.73%	1.18	0.50
*PALBI*	0.710	.000	1.00	86.67%	48.51%	1.68	0.28
*AARC*	0.726	.000	1.00	76.67%	64.36%	2.15	0.36
*CTP*	0.640	.020	2.00	86.67%	40.59%	1.46	0.33
*CTP-Delta*	0.801	.000	0.00	63.33%	78.22%	2.91	0.47
***3-month***							
*MELD*	0.832	.000	27.34	80.43%	82.35%	4.56	0.24
*MELD-Delta*	0.836	.000	2.81	71.74%	89.41%	6.78	0.32
*MELD-Na*	0.850	.000	27.68	89.13%	77.65%	3.99	0.14
*iMELD*	0.840	.000	46.30	86.96%	74.12%	3.36	0.18
*MESO*	0.834	.000	2.10	80.43%	84.71%	5.26	0.23
*ALBI*	0.616	.029	2.00	91.30%	31.76%	1.34	0.27
*PALBI*	0.724	.000	1.00	84.78%	54.12%	1.85	0.28
*AARC*	0.785	.000	1.00	76.09%	71.76%	2.69	0.33
*CTP*	0.653	.004	2.00	84.78%	44.71%	1.53	0.34
*CTP-Delta*	0.750	.000	0.00	56.52%	82.35%	3.20	0.53
***6-month***							
*MELD*	0.832	.000	27.08	78.85%	84.81%	5.19	0.25
*MELD-Delta*	0.792	.000	2.08	67.31%	84.81%	4.43	0.39
*MELD-Na*	0.844	.000	27.08	86.54%	79.75%	4.27	0.17
*iMELD*	0.835	.000	46.30	84.62%	77.22%	3.71	0.20
*MESO*	0.834	.000	1.97	80.77%	84.81%	5.32	0.23
*ALBI*	0.617	.024	2.00	90.38%	32.91%	1.35	0.29
*PALBI*	0.710	.000	1.00	82.69%	55.70%	1.87	0.31
*AARC*	0.771	.000	1.00	73.08%	73.42%	2.75	0.37
*CTP*	0.648	.004	2.00	82.69%	45.57%	1.52	0.38
*CTP-Delta*	0.691	.000	0.00	50.00%	81.01%	2.63	0.62

*P* value <.05 is considered significant.

AUROC, the area under the receiver-operating characteristic curve; PLR, positive likelihood ratio; NLR, negative likelihood ratio; MELD, model for end-stage liver disease; ALBI, albumin-bilirubin; CTP, child-turcotte-pugh score; PALBI, platelet-albumin-bilirubin grade; AARC, APASL ACLF Research Consortium score; MESO, MELD to serum sodium ratio; iMELD, integrated MELD score; MELD-Na, model for end-stage liver disease with serum sodium concentration.
